# Microstructure and Mechanical Properties of Al-Mg-Si Similar Alloy Laminates Produced by Accumulative Roll Bonding

**DOI:** 10.3390/ma14154200

**Published:** 2021-07-27

**Authors:** Zhigang Li, Hao Jiang, Minghui Wang, Hongjie Jia, Hongjiang Han, Pinkui Ma

**Affiliations:** Key Laboratory of Automotive Materials Ministry of Education, School of Material Science and Technology, Jilin University, Changchun 130022, China; lzg@jlu.edu.cn (Z.L.); jianghao18@mails.jlu.edu.cn (H.J.); minghui@jlu.edu.cn (M.W.); jiahj@jlu.edu.cn (H.J.); hanhj@jlu.edu.cn (H.H.)

**Keywords:** Al-Mg-Si alloy, accumulative roll bonding, aging treatment, heterogeneous materials

## Abstract

As the applications of heterogeneous materials expand, aluminum laminates of similar materials have attracted much attention due to their greater bonding strength and easier recycling. In this work, an alloy design strategy was developed based on accumulative roll bonding (ARB) to produce laminates from similar materials. Twin roll casting (TRC) sheets of the same composition but different cooling rates were used as the starting materials, and they were roll bonded up to three cycles at varying temperatures. EBSD showed that the two TRC sheets deformed in distinct ways during ARB processes at 300 °C. Major recrystallizations were significant after the first cycle on the thin sheet and after the third cycle on the thick sheet. The sheets were subject to subsequent aging for better mechanical properties. TEM observations showed that the size and distribution of nano-precipitations were different between the two sheet sides. These nano-precipitations were found to significantly promote precipitation strengthening, and such a promotive effect was referred to as hetero-deformation induced (HDI) strengthening. Our work provides a new promising method to prepare laminated heterogeneous materials with similar alloy TRC sheets.

## 1. Introduction

Aluminum (Al) and its alloys are extensively used in aerospace and automobile industries due to their high specific strength, good formability, and low price [1]. For decades, numerous researchers have attempted to improve the mechanical properties of aluminum alloys, thereby further extending their scope of applications [1,2]. However, an increase in the strength of traditional materials (e.g., Al and its alloys) often comes at the cost of lower plasticity.

As a type of emerging structural material, heterogeneous materials present outstanding performance [3]. Since microstructure formation of dissimilar laminates was controlled by the hetero-deformation between the harder and softer layers, the strength of heterogeneous materials can be improved through the hetero-deformation-induced (HDI) hardening mechanism with almost no loss of plasticity. Therefore, overcoming the limitations of traditional strengthening mechanisms that are difficult to balance between strength and plasticity [3,4]. Accumulative roll bonding (ARB), a traditional technique of severe plastic deformation (SPD), is easily adopted by the industry because it does not need any major modifications to the conventional equipment design [5]. Since the birth of heterogeneous materials, ARB has been used to produce laminated composites [6] and metal-based composites with oxide particles [7,8]. Furthermore, thanks to the development of heterogeneous materials, ARB has been used to simultaneously process two or more different materials and produce laminate composites with special properties, such as Al/Mg [6], Al/Ti [9], Mg/Nb [10], and Cu/Nb [11]. However, dissimilar metals tend to form brittle phases at the interface, which is not conducive to good bonding. Besides, the composites made from dissimilar metals are difficult to recycle. As a result, heterogeneous laminate materials composed of similar metals are worthy of further study.

Various metal materials of the same base metals have been used to produce laminate composites, such as AA6061/AA5754 [12], AA7075/AA1100 [13], and Al–Li/Al–Li–Zr [14]. V. G. Arigela et al. [15] produced AA2014/AA6063 composites by ARB and compared the deformation processes in individual layers. However, the strength of the composites was found to drop slightly because they did not undergo aging treatments, and the differences between layers were not maintained. Lihong Su et al. [16] produced an AA1050/AA6061 composite sheet with ultrafine-grained (UFG) structure by a five-cycle ARB procedure. They revealed that the strength from grain boundaries had little effect on the aluminum alloy. Geng et al. [17] combined Al-Mg-Si alloy with Al-Mg-Si/SiC composites to produce a novel laminate composite by a five-cycle ARB procedure and subsequent aging. They realized an improved yield strength (∼300 MPa) with no loss in elongation, and they attributed such a phenomenon to bimodal-sized $\beta^{\prime \prime }$ precipitates as a result of the layered distribution of TiC nanoparticles. Their research provided a promising strategy to produce aluminum laminates from similar materials.

Aside from easy recycling, similar laminates are very advantageous in numerous aspects in actual production procedures compared with dissimilar laminates, but few studies have investigated the microstructural evolution and mechanical properties of ARB-processed similar laminates. In this work, twin rolled Al-Mg-Si alloy fabricated with different TRC parameters were used as the starting materials to produce similar laminates. These laminates were observed for their mechanical properties and microstructure evolution via scanning electron microscopy (SEM) and electron backscatter diffraction (EBSD). As the Al-Mg-Si alloy is age-hardenable and capable to form various precipitates upon aging, transmission electron microscopy (TEM) was utilized to observe the precipitates after aging treatment.

## 2. Experiment

TRC has been attracting attention because of its low production cost [18]. Besides, TRC sheets with different structures and properties can be fabricated by changing the TRC parameters [19,20], but the quality of TRC strips is always a concern. Center segregation, which appears in the final solidified zone of the TRC strip, is one of the most typical and inevitable microstructural casting defects in a TRC strip [21,22]. Laminates can unify the structure on the macro scale, thereby enhancing the structural uniformity and improving the mechanical properties of the sheets.

**Figure 1:**
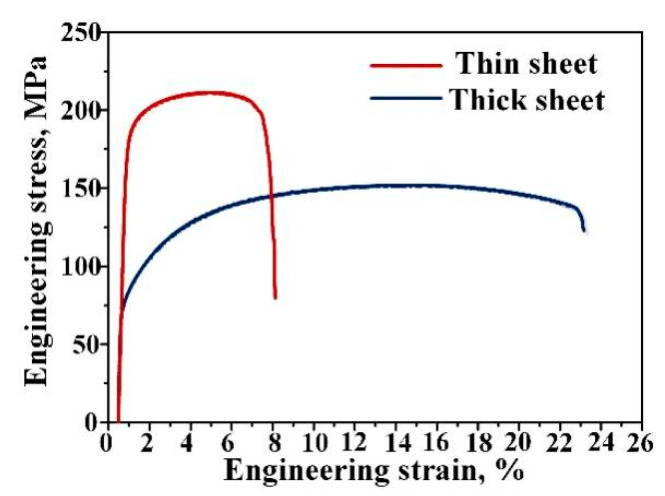
The engineering stress–strain curves of TRC sheets.

In the present study, the starting materials were fabricated with different TRC parameters. TRC Al-Mg-Si alloys, whose detailed chemical compositions are given in Table 1, were used for ARB processing without prior treatment. The parameters of TRC processing for the thick sample were as follows: a casting temperature of 690 °C, a rolling speed of 8 m/min, a roller gap of 2.25 mm, and a sheet thickness of 3 mm. When processing the thin samples with TRC, the parameters became 700 °C, 10 m/min, 0.3 mm, and 2 mm for casting temperature, rolling speed, roller gap, and sheet thickness, respectively. The tensile properties of TRC sheets are shown in Figure 1.

Before the TRC Al-Mg-Si sheets were processed with ARB, their contact surfaces were cleaned with a wire brush and degreased with acetone to ensure smoothness and flatness. Subsequently, the sheets were stacked and wired together to achieve good bonding between them.

In route 1, the stacked sheets were preheated for 10 min at 300 °C and rolled to reduce their thickness by half without lubricants. Then the sheets for ARB processing were cut in half and stacked to the initial thickness, and an identical procedure was repeated up to three cycles to obtain the laminates. Finally, the laminates underwent solution treatment at 550 °C for 30 min and aging treatment at 175 °C for 6 h.

**Figure 2:**
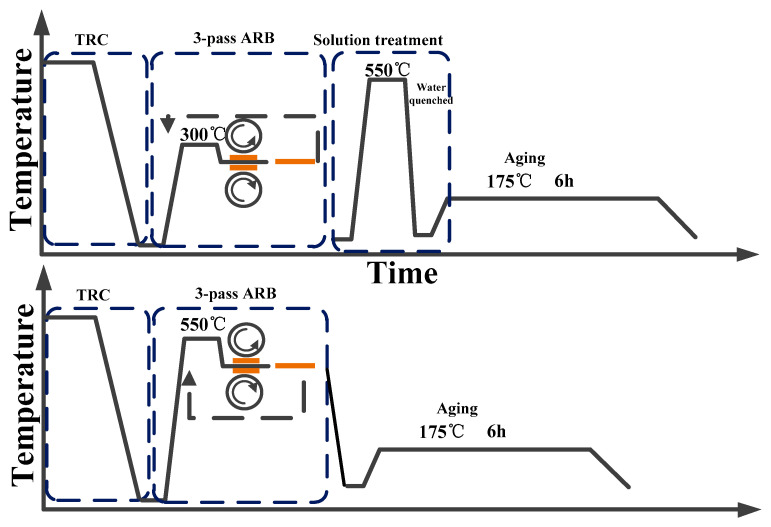
Thermo-mechanical route of this study.

In route 2, the stacked sheets were preheated for 30 min at 550 °C and rolled to reduce their thickness by half and obtain laminates. The laminates were subject to aging treatment at 175 °C for 6 h, and no solution treatment was performed. The schematic illustration of preparation process is shown in Figure 2.

The microstructure was investigated with a field emission SEM (ZEISS EVO18, Oberkochen, Germany) equipped with an energy dispersive spectroscopy (EDS) and an EBSD detector and a TEM (JEOL, Tokyo, Japan) with an accelerating voltage of 200 kV. Samples for EDS observation were prepared by traditional grinding and polishing. Specimens for EBSD observation were prepared by mechanical polishing for SEM characterization, followed by electro-polishing (60 s, 20 V, Naibo, Shanghai, China) in perchloric acid solution (10 mL HClO_4_, 90 mL alcohol). EBSD data were analyzed using Channel 5. TEM samples were sliced from the sheets along the RD-TD plane. After that, the TEM slices were ground to ~40 μm in thickness and punched into disks with a diameter of 3 mm by a Gatan punching machine (Naibo, Shanghai, China). Next, these disks were further thinned by electro-polishing with a twin-jet electro-polisher. Room temperature tensile tests were conducted on an electronic testing machine (SHIMADZU, Suzhou, China) with a video extensometer (SHIMADZU, Suzhou, China), and the tensile direction was parallel to the rolling one. The initial strain rate was 1 × 10^−4^ s^−1^.

## 3. Results and Discussion

### 3.1. Microstructure Characterization

Generally, in a composite, metal co-deformation is difficult if the metals differ greatly in their flow stresses and hardness [15]. The harder material is generally more susceptible to necking and rupturing so that the final microstructure is always full of fragments of the harder material in its softer matrix, rather than a continuous laminated structure. However, in this study, the interfaces formed between layers during each cycle were straight without obvious cracks, which could be attributed to high deformation temperature and good plasticity of the material. Therefore, we suspected that similar layers share similar flow behaviors, leading to good atomic bonding at the interfaces during ARB.

**Figure 3:**
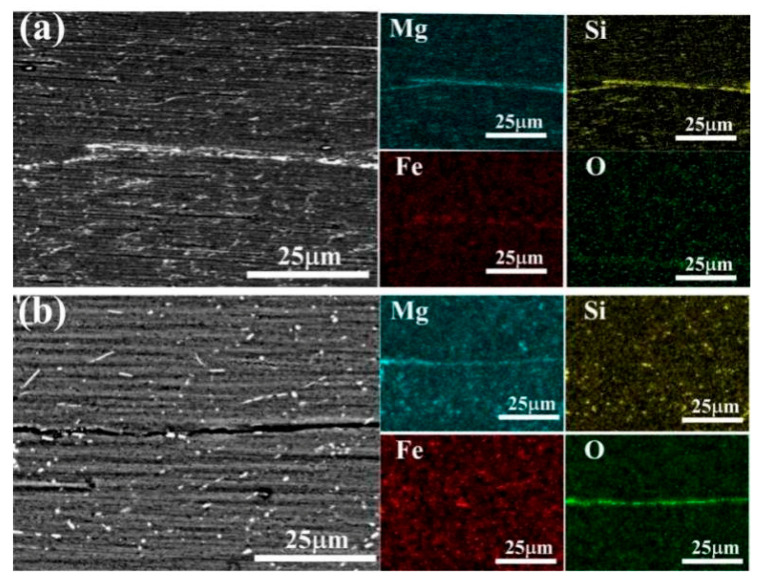
Surface scan energy spectrum of ARB sheets interface, (**a**) 300 °C, (**b**) 550 °C.

Figure 3 shows the surface SEM spectra of ARB sheet interfaces fabricated at different temperatures. It can be inferred from the figures that solute elements (Mg, Si, and Fe) exhibited obvious aggregation at the interfaces. Besides, a continuous and semi-continuous second phase containing Al, Mg, Si, and Fe was formed during the ARB processing at 300 °C. However, when processed with ARB at 550 °C, the interfaces only revealed the aggregation of Mg, indicating that the diffusion and aggregation of Mg occurred while Si and Fe were not significantly enriched near the interface. The alloy surface is susceptible to oxidation at high temperatures, and when the temperature rises above 394 °C, Mg atoms become significantly more mobile [12], making them much easier to combine with O atoms and form oxides.

**Figure 4:**
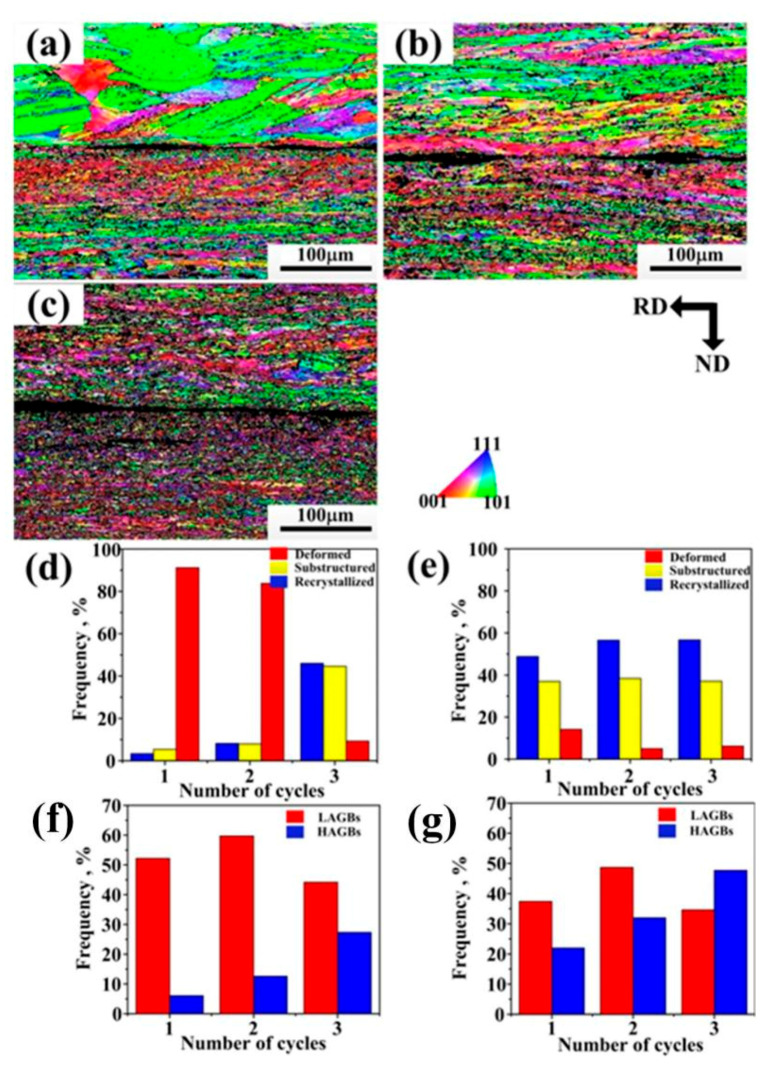
EBSD inverse pole figure (IPF) maps of the ARB sheets (**a**) after one cycle, (**b**) after two cycles, (**c**) after three cycles; (**d**) recrystallization map fraction of the thick sheet; (**e**) recrystallization fraction map of the thin sheet; (**f**) grain boundary maps of the thick sheet; (**g**) grain boundary maps of the thin sheet.

EBSD characterizations were performed on the ARB samples to understand the microstructural evolution. Figure 4 shows the laminates produced by route 1, from which the transition from coarse to ultrafine-grained microstructures from one side to the other could be observed on both two sides. As the cycles continued, deformations became gradually uniform. The grains of the thick sheet were elongated. Besides, they started to form substructures, creating fine-grained structures after three cycles with an average grain size of 3.6 μm. The grains of the thin sheet recrystallized after the first cycle, indicating major grain fragmentation on the thin sheet (Figure 4a), and the average grain size of recrystallized grains was about 2.9 μm. Grain refinement occurred in the subsequent deformation. After three cycles, typical ultrafine grains were present on both sides, suggesting major grain fragmentation on the thick sheet (Figure 4c). The percentage of high-angle grain boundaries (HAGBs) in the thick sheet after three cycles was higher than that in the thin sheet.

**Figure 5:**
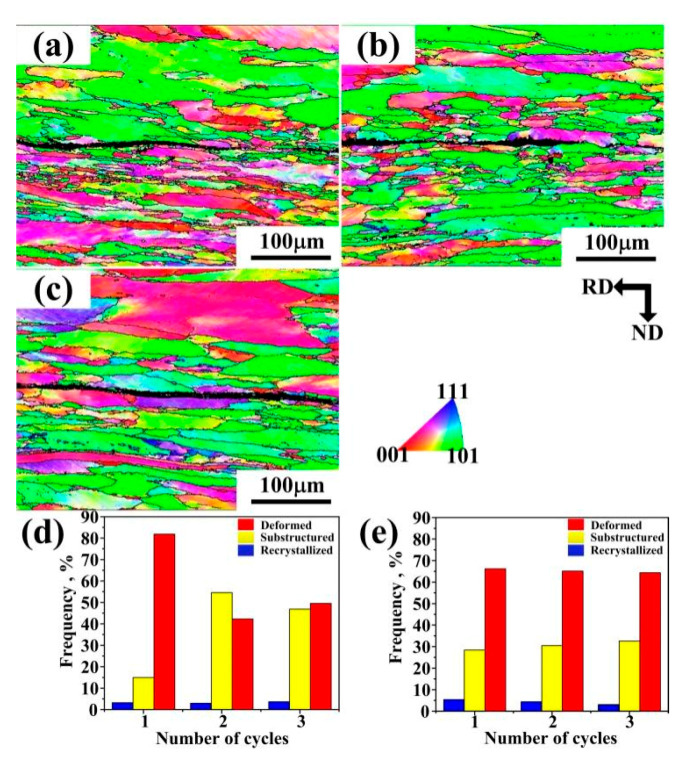
IPF maps of ARB sheets (**a**) after one cycle, (**b**) after two cycles, (**c**) after three cycles; (**d**) recrystallization fraction in the thick sheet; (**e**) recrystallization fraction in the thin sheet.

Figure 5 is the EBSD map of the laminates produced by route 2. The deformed grains experienced quick static recovery and recrystallization before ARB processing. Although deformation occurred in high temperatures, the deformed structure, and sub-structures were still dominant in the ARB sheets, while the recrystallized grains accounted for only a small proportion. The sub-structures existed because deformation at higher temperatures would cause dislocation rearrangement, which lowered the dislocation density ($\sim 7.3{\;\times \;10}^{14}{\;m}^{-2}$) and limited the formation of sub-grain boundaries. As ARB processing continued, the grains elongated in RD and the fraction of sub-structures increased.

### 3.2. Mechanical Properties

**Figure 6:**
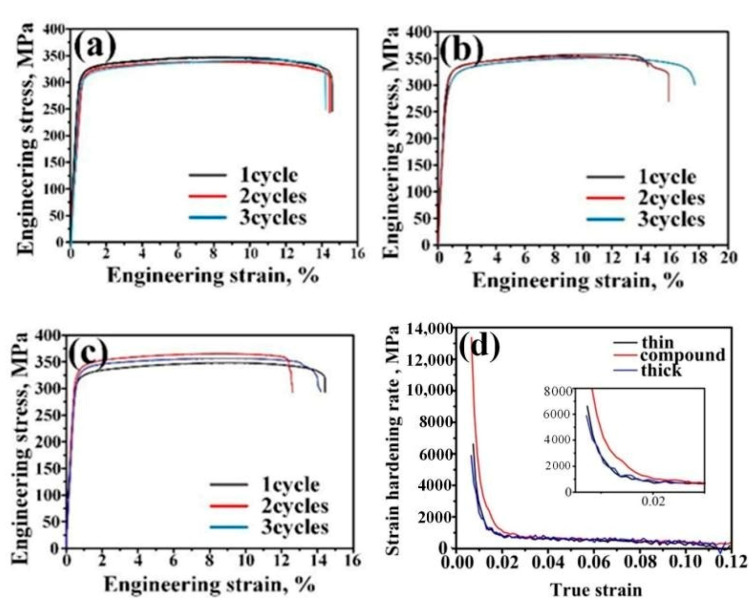
Engineering stress–strain curves of 300 °C ARB sheets after solution and aging treatment: (**a**) thin sheets, (**b**) compound sheets, (**c**) thick sheets, and (**d**) strain hardening rate of different sheets after three-cycle ARB processing at 300 °C.

The stress–strain curves of the ARB sheets after solution and aging treatments are displayed in Figure 6. Compound ARB sheets exhibited better ductility after three cycles, and their yield strength (YS) and ultimate tensile strength (UTS) values were 297 and 342 MPa, respectively, with an elongation percentage of 18%. With almost no reduction in strength, the compound ARB sheets showed 28.6% higher elongation than the other two sheets. The satisfactory comprehensive mechanical properties demonstrate the significant enhancement and superiority of compound ARB sheets over the other two sheets. Additionally, Figure 6d illustrated that the compound sheets after a three-cycle ARB processing exhibited larger strain hardening rates than similar ARB sheets.

**Figure 7:**
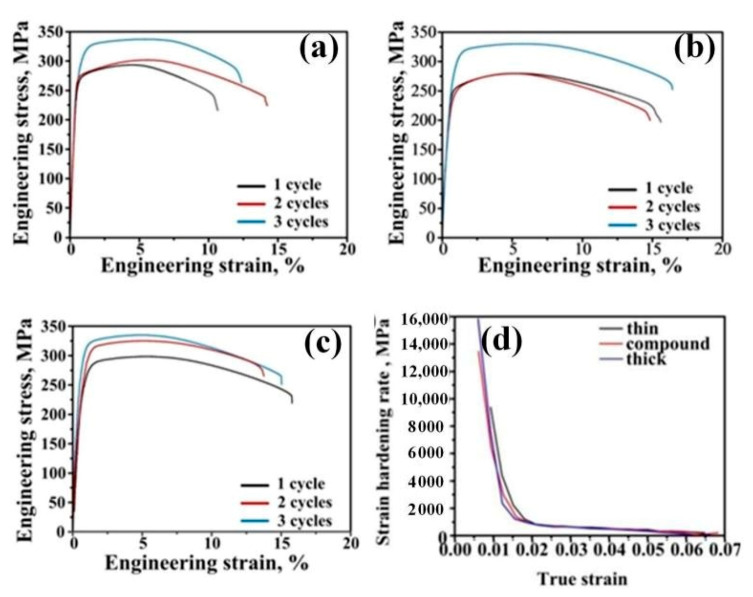
Engineering stress–strain curves of 550 °C ARB sheets after aging treatment: (**a**) Thin sheets, (**b**) compound sheets, (**c**) thick sheets, and (**d**) strain hardening rate of different sheets three-cycles ARB processing at 550 °C.

The strength of the Al-Mg-Si alloy is controlled by the precipitates and strain-hardening behaviors of the alloy. After deformation and immediate water quenching, the laminates underwent aging treatment directly without solution treatment. The YS and UTS values of the compound ARB sheets were 319 and 342 MPa, respectively, and the elongation rate was 15%. Besides, the samples processed by a three-cycle compound ARB revealed comparable strain hardening rates to the two similar ARB sheets (Figure 7d).

### 3.3. Microstructural Variations

**Figure 8:**
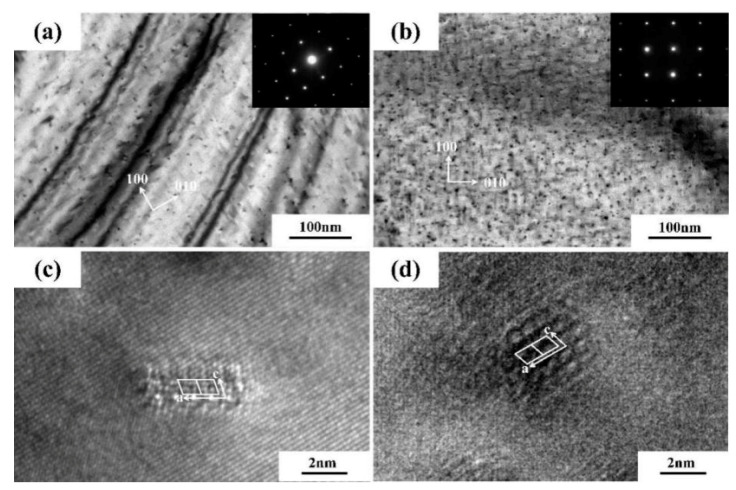
TEM micrographs of the ARB composite sheets alloys: (**a**) Thin sample, (**b**) thick sample, and (**c**,**d**) the corresponding HRTEM figures of *β*^″^ precipitates.

The TEM micrographs depicting the distribution of intragranular precipitates in the compound ARB sheets are shown in Figure 8. Judging from the high-resolution TEM (HRTEM) diagrams (Figure 8c,d), the precipitated phase was the β” phase. However, the precipitated phases showed varied sizes and number densities in different areas on both sides.

All the TEM images were viewed along the <001> Al direction, and the precipitate number density can be expressed as Equation (1) [23]:

(1) $$P=\frac{2N}{AL}$$

where N is the number of needles in the zone-axis direction, A is the respective area on the TEM image, and L is the mean particle length.

The volume fraction of a precipitate can be expressed as Equation (2) [24]:

(2) V=PLS

where S is the mean average cross-sectional area of particles.

Table 2 shows the quantitative measurement results. Compared with the thick sheet, the thin sheet contained more refined and a greater number of precipitates, but the number density of precipitates dropped in the thin sheet. Such phenomena indicated that rapid cooling during TRC can refine the precipitates and lead to an increase in the number density of them.

The macroscopic properties of the alloys reflect the coupling effects of microstructures. Firstly, the yield strength σ can be expressed as Equation (3) [25,26]:

(3) $${\sigma =\sigma }_{0}{+\sigma }_{B}{+\sigma }_{D\;}{+\sigma }_{p}$$

where ${\;\sigma }_{0}$ is the strength of the metal matrix, $\sigma_{B}$ is strength of the grain boundaries, $\sigma_{D}$ is the strength of the dislocation, $\sigma_{p}$ is the strength of the precipitate.

The contribution of grain boundaries is usually calculated by the Hall–Petch relationship Equation (4) [25]:

(4) $$\sigma_{B}{=k}_{y}d^{-\frac{1}{2}}$$

which d is the grain diameter, and the $k_{y}$ value in the aluminum alloy is very small, which is 0.068 MPam^1/2^. The difference of grain size between different ARB sheets is small, and has little effect on the yield strength, so it is not considered in the experiment.

Dislocation strengthening is generally calculated using Equation (5) [27]:

(5) $$\sigma_{D}{=M\alpha Gb\rho }^{-\frac{1}{2}}$$

where M is the Taylor’s factor (*M* = 3.06 for aluminum alloy), α = 0.2 in aluminum alloy; G is shear modulus (G = 27 GPa in aluminum alloy); b is the Burgers vector ($b=2.86{\;\times \;10}^{-10}\;m$); ρ is the dislocation density, which can be calculated using Equation (6) [28]:

(6) $$\rho =\frac{2\theta }{\mu b}$$

where θ is the local misorientation (<3°), and μ is the unit length (step size, 0.7 μm). For the ARB sheets in route 1, the dislocation densities are very low and almost close. The dislocation strengthening is negligible.

For the ARB sheets in route 2, the dislocation strengthening of different ARB sheets after three cycles is 126, 126 and 102 MPa, respectively.

In this study, the precipitate strengthening is a key factor, and is generally calculated using Equations (7) and (8) [2,29],

(7) $$\sigma_{p}=\frac{M{\bar{F}}_{\beta^{\prime \prime }}}{bL_{\beta^{\prime \prime }}}$$

(8) $$L_{\beta^{\prime \prime }}={\left(\frac{2\pi }{V_{\beta^{\prime \prime }}}\right)}^{1/2}r_{\beta^{\prime \prime }}$$

where ${\bar{F}}_{\beta^{\prime \prime }}$ is the mean stress induced by the *β*^″^ precipitate distribution; $L_{\beta^{\prime \prime }}$ is average spacing of *β*^″^ precipitates on the slip plane; $V_{\beta^{\prime \prime }}$ and $r_{\beta^{\prime \prime }}$ are the volume fraction and radius of *β*^″^ precipitates. Specifically, ${\bar{F}}_{\beta^{\prime \prime }}$ can be described as Equation (9) [2,29]:

(9) $$\begin{array}{l} {\bar{F}}_{\beta^{\prime \prime }}=kGb\{\frac{K{∆}^{2}}{2}[exp(-\frac{{\bar{r}}_{\beta^{\prime \prime }}}{{∆}^{2}})-exp(-\frac{\left(r_{c}-{\bar{r}}_{\beta^{\prime \prime }}\right)}{{∆}^{2}})]+\\ \frac{K∆{\bar{r}}_{\beta^{\prime \prime }}\sqrt{\pi }}{2}[erf(\frac{{\bar{r}}_{\beta^{\prime \prime }}}{∆})+erf(\frac{r_{c}-{\bar{r}}_{\beta^{\prime \prime }}}{∆})]\}+kGbr_{c}\{\frac{K∆\sqrt{\pi }}{2}[1-erf(\frac{r_{c}-{\bar{r}}_{\beta^{\prime \prime }}}{∆})]\} \end{array}$$

where k is a constant and $k=\frac{b}{r_{c}}$, ∆ is the standard deviation of the radius distribution and $\frac{∆}{{\bar{r}}_{\beta^{\prime \prime }}}$; $r_{c}=3.0\;\mathrm{nm}$ is the radius of the shearables to transform into non-shearables, and it can be described as Equation (10) [2]:

(10) $$K=\frac{2}{∆\sqrt{\pi }(1+erf(\frac{{\bar{r}}_{\beta^{\prime \prime }}}{∆}))}$$

The precipitate strengths in the thick and thin sheets were calculated to be about 201 and 237 MPa, respectively. The strength of the ARB sheets was predominantly determined by the precipitates, while grain boundary strengthening and dislocation strengthening were of minor importance as a result of solution treatment. Consequently, the differences of the precipitates between the two sides were reflected as the soft and hard domains during tensile deformation, and the mutual constraint between the soft and hard lamellae produced HDI hardening effects.

In route 2, the strength of the ARB sample was governed by precipitates, and dislocation strengthening (~100 MPa) played a minor role. Inter-cycle annealing led to periodical dislocation annihilation, but the dislocation density was almost constant because the reduction and deformation temperatures during ARB processing were almost the same. Aside from the two mechanisms mentioned above, dispersion strengthening, which is closely related to the phase size, should be considered as an influential factor to the sample strength. However, the size of secondary phases was large, leading to weak dispersion strengthening. More specifically, dispersion strengthening almost disappeared when the particle size grew to the micron level. Dynamic precipitation at high temperatures would not result in a rise in strength; instead, it would weaken solution strengthening effects of Mg and Si solutes [30]. Besides, dislocations are known to attract solutes in supersaturated solid solutions and thereby impact precipitation. Such a phenomenon would reduce the content of solutes in the matrix and weaken the driving forces of homogenous nucleation, which could be an explanation of the lower sample strengths after artificial aging [31].

## 4. Conclusions

In this work, sheets of heterogeneous material laminates made from similar metals were successfully fabricated by ARB at 300 °C. Solution treatments and hot rolling were administered together to shorten the processing time. This work provides a new solution in the preparation of heterogeneous material laminates, and the conclusions are as follows:

(1) The two TRC sheets responded differently to the imposed deformation during ARB processing at 300 °C, which could be explained by the differences of starting materials in terms of their microstructures and mechanical properties.
(2) When conducting ARB processing at 300 °C, a large number of solute atoms aggregated to form intermetallic compounds at the interface. On the other hand, most Mg atoms aggregate during ARB processing at 550 °C.
(3) The phases and distribution of precipitates varied because of the differences in the starting materials. Consequently, the two sides of the sheets differed as the soft and hard domains during tensile deformation, leading to greater strain hardening effects. Finally, the heterogeneous material laminates manufactured in this study exhibited better comprehensive mechanical properties.

## Figures and Tables

**Table 1:** The composition of TRC alloy (wt.%).

Si	Fe	Mg	Mn	Cu	Al
1.4–1.5	0.1	0.9–1.0	<0.01	<0.01	Bal.

**Table 2:** Quantitative measurements of the particle size of *β*^″^ precipitates in compound ARB samples.

Sample	Average cross Section Area/nm^2^	Average Radius/nm	Number Density/nm^3^	Volume Fraction/%
Thin sheet	9.6	8.2	1.1 × 10^−3^	8.7
Thick sheet	14.5	20.3	2.1 × 10^−4^	6.2

## Data Availability

Data sharing not applicable. No new data were created or analyzed in this study. Data sharing is not applicable to this article.
